# Barriers and enablers to general practitioner referral of older adults to hearing care: a systematic review using the theoretical domains framework

**DOI:** 10.1007/s41999-024-01124-5

**Published:** 2025-05-22

**Authors:** E. C. Davine, P. A. Busby, S. Peters, J. J. Francis, J. Z. Sarant

**Affiliations:** 1 The University of Melbourne, 550 Swanston Street, Carlton, VIC 3053 Australia; 2 Ottawa Hospital Research Institute, Ottawa, Canada

**Keywords:** Primary care, Referral, Audiology, General practice, Theoretical domains framework, Hearing care

## Abstract

**Purpose:**

The purpose of this systematic review was to identify and synthesise the literature regarding barriers and enablers affecting general practitioner (GP) referral to hearing care for their older patients (50 years and over).

**Methods:**

A search of peer-reviewed articles reporting primary empirical studies was conducted across CINAHL, Ovid Medline and Scopus, with search terms relating to the search domains “General Practitioner”, “Referral”, “Hearing loss”, and “adults aged 50 and older”. Qualitative and quantitative studies were included if they reported barriers or enablers to referral. A mixed-methods approach was used to synthesise the findings of the included studies, firstly into the Theoretical Domains Framework of behaviour change, and then into more granular sub-themes.

**Results:**

The initial search yielded 859 unique studies. Title and abstract screening identified 21 studies of possible relevance, and full text review identified seven studies for inclusion in this review. A total of 19 unique themes were identified and coded to 10 of the 14 domains of the Theoretical Domains Framework; however thematic overlap between studies was low and fewer than half of these themes were consistently identified as either a barrier or enabler. Four main barriers to referral to hearing care were identified: Lack of time, lack of familiarity with diagnostic criteria and tools, lack of knowledge of treatments and higher relative importance of other health conditions.

**Conclusion:**

The minimal overlap of themes and low agreement on which of these constitute barriers and enablers for referral indicates a need for further research to provide greater clarity in this area and explain the heterogeneity of these results.

**Supplementary Information:**

The online version contains supplementary material available at 10.1007/s41999-024-01124-5.

## Key summary points

**Aim:**

To explore the barriers and enablers affecting General Practitioner referral of older adults to hearing care that have been reported in the published literature.

**Findings:**

This review identified four barriers and five enablers to hearing care referral. However, minimal overlap in thematic content, contradictory findings between studies and a low number of total published studies in this area suggest that data saturation has not been reached.

**Message:**

Further research is required to understand the heterogeneity of findings observed in this review.

**Supplementary Information:**

The online version contains supplementary material available at 10.1007/s41999-024-01124-5.

## Introduction

Presbycusis (age-related hearing loss) affects approximately 65% of people aged 60 or older, with incidence increasing steadily from the age of 50 [1]. The 2019 Global Burden of Disease study identified hearing loss as the third most common cause of years lived with disability among people aged 70 and over [2]. Apart from the immediate impacts to speech intelligibility and communication, age-related hearing loss is associated with significant comorbidity and impacts on quality of life, including increased rates of anxiety, depression and social isolation, faster rates of cognitive decline, an increased risk of falls, and greater overall mortality [3–8].

Despite these well-established co-morbidities of untreated hearing loss and the efficacy and safety of existing treatments such as hearing aids, hearing loss in older adults remains under-diagnosed and under-treated [9, 10]. Amongst adults who have noticed a hearing issue, 32% have never consulted a clinician regarding their hearing, and 28% have never had any form of hearing test [11]. These low rates of self-driven help-seeking among adults with hearing loss lead to corresponding delays in treatment, with an average delay between becoming eligible for hearing aids and being fitted of 8.9 years [12].

While audiology clinics are commonplace in the developed world, their standalone nature means that they are typically reliant on either: (a) Self-motivated hearing help-seekers, who identify a hearing problem and independently seek out assessment and treatment, or (b) Advice to patients (whether targeted or routine) from other healthcare providers who are in more regular contact with older adults such as a primary care general practitioner (GP).

On average, Australians visit a GP 6 times per year, and 87% of the population visit their GP at least once in a 12-month period [13]. It is estimated that adults aged 50 and over account for nearly 50% of all GP consultations; yet only 0.3% of all GP consultations involve actions, referrals or discussions related to hearing loss [14]. This high degree of contact with the age group most at risk for presbycusis ideally positions GPs to refer older adults for hearing care. Thus, the current lack of GP referrals to audiologists represents an evidence-practice gap.

Reasons for evidence-practice gaps vary across healthcare settings, and there is no one-size-fits-all approach for rectifying them [15]. The field of Implementation Science was developed in response to the need to build a reliable evidence base for implementing evidence into real-word clinical practice [16]. Central to implementation science is the use of theories, theoretical frameworks and models to approach data in an empirical, structured and consistent manner, in order to reduce the role of implicit biases and assumptions [17].

One such framework, the Theoretical Domains Framework (TDF) was developed to identify and subsequently address factors which may influence healthcare provider behaviour [15]. The TDF consists of 14 domains relating to potential barriers and enablers (Table, online resource 1) which may affect the uptake of a desired evidence-based practice [18, 19].

Not all domains will be relevant to every setting, but by making use of the TDF, data can be systematically coded into these domains, allowing for targeted implementation strategies to be developed to address the modifiable barriers and enhance the enablers that are identified as impacting evidence-based practice adoption [20].

The aim of this systematic review was to identify and synthesize the potential barriers and enablers to GP audiology referral for adults aged 50 years and over following the TDF. To the best of our knowledge, this is the first systematic review to be conducted in the area of referral from GP primary care to audiology.

## Methods

### Search strategy

A detailed protocol for this systematic review was registered in PROSPERO (CRD42023412427), and a checklist of the Preferred Reporting Items for Systematic Reviews and Meta-Analyses (PRISMA) can be found in Table, Online Resource 2. Literature searches were conducted across CINAHL, Ovid MedLine and Scopus on 25th January 2023, and were repeated on 6th August 2024 to ensure all relevant studies were identified.

The search terms for the review were chosen to reflect the following four key search domains: general practitioner, referral, hearing loss, and the target population of adults aged 50 and older (See Table, Supplementary Digital Content 3 for full search strategies for each database). An inverse search was performed on the reference lists of all studies identified for data extraction.

### Inclusion and exclusion criteria

Inclusion criteria were categorised using the SPIDER framework (Sample, Phenomenon of Interest, Design, Evaluation, Research type) [21]. Studies were eligible for inclusion if they used a *sample* of general practitioners/family doctors/primary care doctors. If multiple demographics were included in the participant pool, GP data was required to be reported separately to data from non-target groups (e.g. patients, nursing staff, or medical professionals not working with the target age group such as paediatricians). The *Phenomenon of Interest* was GP views or opinions about referral to audiology. The *Design* of each study influenced how data was extracted and analysed. For *Evaluation*, we included data which categorised GP perspectives on referring for hearing care in adults aged 50 and older. For *Research type*, we included both qualitative (e.g., interviews, focus groups, written responses) and quantitative studies (e.g., surveys). Only primary, empirical studies were included.

No language restrictions were placed on the initial search, though data extraction was only performed on those available in English.

No limits on publication date were set, with all studies published up to the time of searching considered. As the body of published literature on this topic was anticipated to be small, it was unclear whether quantitative studies conducted to date fully encompassed the experiences of the GP, or whether they were restricted by survey/question design based on what *researchers* felt were important influences on GPs’ clinical decisions. Therefore, while an overall convergent approach to data integration was taken, attention to any thematic discrepancies between qualitative and quantitative studies was included as part of this review.

### Screening

Initial screening of data was carried out in Covidence Systematic Review Software (Veritas Health Information, Melbourne, Australia. Available at www.covidence.org). Abstract and title screening was carried out by two researchers independently (ED and IH), with conflicts resolved by discussion until consensus was reached. Full text screening was completed initially by one author (ED), with critical review conducted by a second author (PB).

### Quality assessment

Quality assessment of the included studies was carried out independently by two authors (ED and PB), with any conflicts resolved by discussion until consensus was reached. The Quality Assessment with Diverse Studies (QuADS), a tool designed for use in mixed-methods reviews, was used to assess the quality of included studies [22]. The QuADS consists of 13 criteria, each of which are scored from 0 (criterion not mentioned at all) to 3 (criterion explicitly addressed). Due to the diversity of methods the QuADS is designed to encompass, no pre-determined cut-off score for high- or low-quality studies is given; rather, quality is addressed descriptively for each study.

Due to the anticipated small number of studies, quality assessment was intended not as a tool for excluding those studies deemed to be of low-quality, but instead for considering the validity of findings.

### Data extraction

Data Extraction was undertaken by two authors independently (ED and PB), with conflicts resolved by consensus. As this was a mixed methods review, three different types of data were extracted for analysis:

1. Questionnaire items
2. Quantitative data i.e., questionnaire item responses
3. Qualitative data i.e., participant quotes or author summary statements which included direct reference to thematic content

A well-established method for combining qualitative and quantitative data for analysis is the convergent approach [23]. Under this method, one class of data is transformed into the other; i.e., quantitative data is transformed into qualitative data (qualitisation) or vice-versa (quantification). This allows for the integrated data to be interpreted together, creating a fuller picture than would be possible by interpreting each type of data separately. The Joanna Briggs Institute Mixed Methods Review Methodology Group recommends the qualitisation of data, rather than quantification when undertaking data integration, as ascribing numerical values to qualitative data is more prone to error than is describing quantitative data narratively [24].

**Fig. 1:**
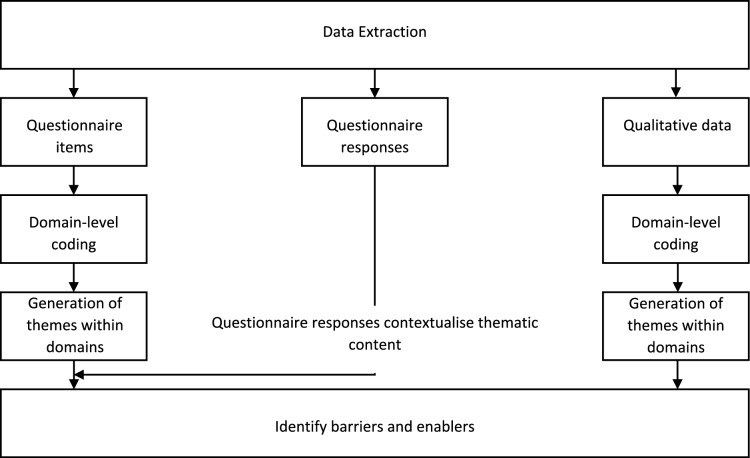
Data transformation and convergence process

As illustrated in Fig. 1, questionnaire items and qualitative data contributed thematic content. Quantitative data (i.e., questionnaire responses) served to contextualise the questionnaire items into the categories of “barrier” or “enabler”.

Questionnaire items were considered to contain one form of data in their thematic content, as they reflect only a concept proposed by a researcher, rather than a stance taken by a participant. To contextualise this content, participant stances were derived from the responses to questionnaire items, with the response from the majority of participants used to determine their barrier or enabler status. For example, for the question “Does hearing loss negatively affect older persons’ quality of life?”, we would consider a majority disagree response to place this as a barrier, as it would demonstrate low knowledge of the impacts of hearing loss, which could be inferred to act as a barrier to referral, as GPs may not see it as an important health consideration. Participant quotes were considered to contain two kinds of data: thematic content, and the participants’ stance regarding this content (i.e., whether they viewed the content as a barrier or an enabler to referral).

### Data analysis

Following data extraction, all questionnaire and qualitative items were collated into a spreadsheet by one author (ED). These were then coded into the 14 domains of the Theoretical Domains Framework (TDF) and reviewed by a second author with extensive experience in using this framework (JF), who provided critique and validation of the coding decisions. Disagreements were discussed until consensus was reached.

Once consensus was reached on the appropriate domain for each item, thematic analysis was undertaken inductively to identify themes within each TDF domain [25]. This was carried out by one author (ED), with critique and a consensus process conducted by a second researcher (JF) as described above. No set number of themes per domain was defined before undertaking this process, with the number and content of themes determined wholly by the content in each domain.

## Results

**Fig. 2:**
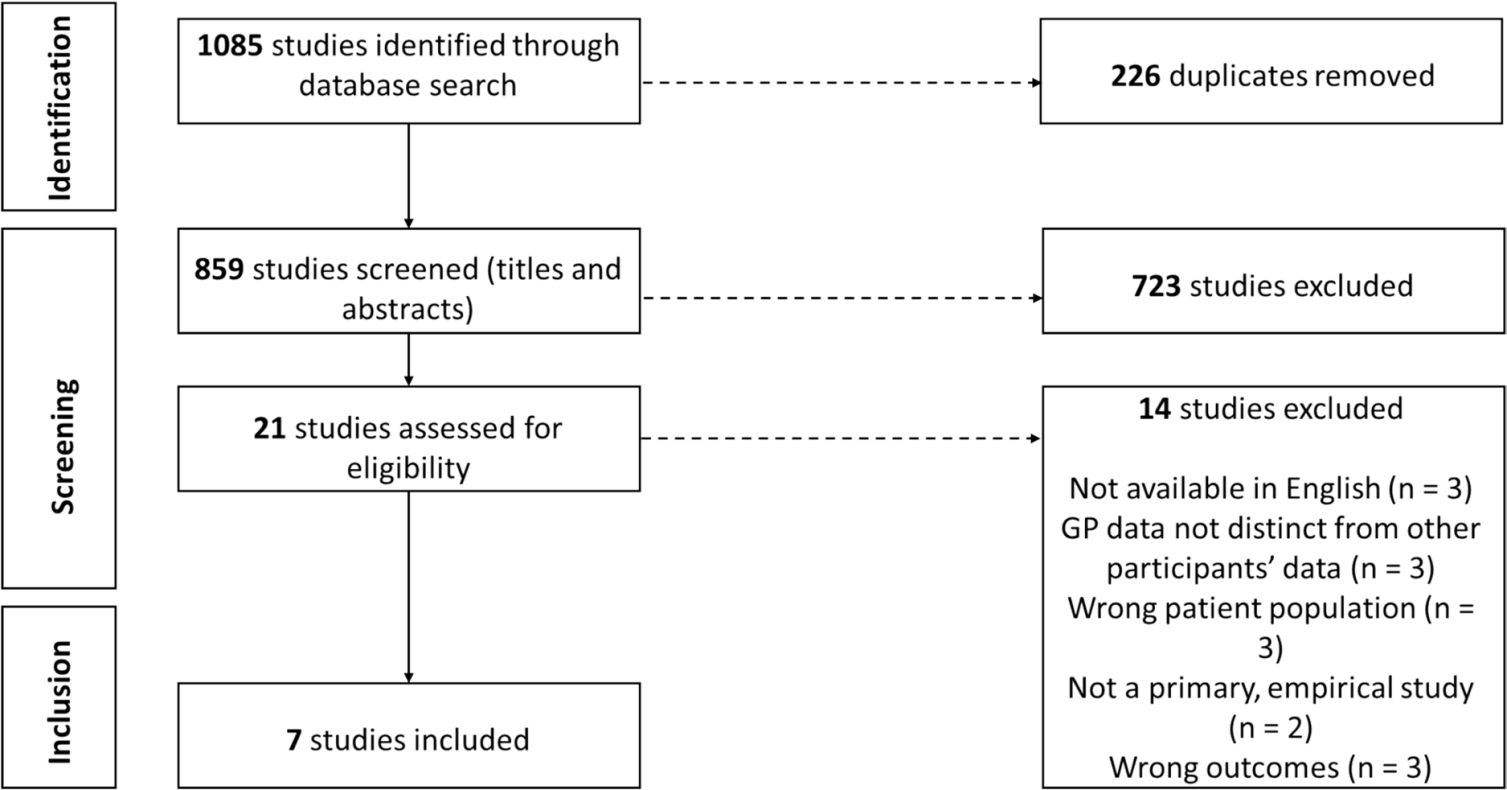
PRISMA flow diagram

After removal of duplicates, 859 studies were identified for eligibility screening. Abstract and title screening yielded 21 studies of possible relevance, and full-text screening identified a total of 7 studies for data extraction. Inverse searching of the reference lists of these studies identified no further studies of relevance beyond those already found in the initial search. Figure 2 shows a PRISMA flow diagram of preferred reporting items for systematic reviews and meta-analyses.

Key features of the 7 included studies are listed in Table 1. The majority of studies were cross-sectional and conducted in the USA.

**Table 1:** Key features of included studies

References	Year of publication	Country	Study design	No. participants	Characteristics of participants
Parving et al. [26]	1996	Denmark	Cross-sectional questionnaire study	42	GPs in Copenhagen who saw patients aged 80 and older
Cohen et al. [27]	2005	USA	Cross-sectional questionnaire study	85	Primary Care Practitioners (PCPs) in Kentucky and Tennessee
Danhauer et al. [28]	2008	USA	Cross-sectional questionnaire study	32	PCPs in Santa Barbara, California
Johnson et al. [29]	2008	USA	Cross-sectional questionnaire study	95	PCPs who practiced within one mile of the centre of the 25 most populous U.S. cities
Gilliver & Hickson [30]	2011	Australia	Cross-sectional questionnaire study	110	Australian medical practitioners: excluding those who identified as having no contact with patients 60 and older
Zazove et al. [31]	2017	USA	Qualitative interview study	23	Family medicine practitioners at two clinics in Michigan
Sydlowski et al. [32]	2022	USA	Cross-sectional questionnaire study	406	US healthcare providers (% of patients aged 50 and older asked in questionnaire)

Quality judgements across the 13 criteria from the QuADS were mixed for all studies (see Table, Supplementary Digital Content 3 for full quality judgements for each study), with no single study being judged as consistently high quality.

Criterion 12 (*evidence that the research stakeholders have been considered in research design or conduct)* was generally the lowest scoring criterion in this review; five of the seven studies included no mention of this at all*.* Two studies, Johnson et al. [29] and Danhauer et al. [28], used a questionnaire tool which was “developed through an exhaustive iterative process using input from physicians, colleagues, and experts in the field”. While the process by which this tool was developed was not described, this was the only mention of physician involvement in research design across the 7 studies.

Johnson et al. [29] and Danhauer et al. [28] used the same questionnaire across both studies, therefore thematic data was considered to be identical. For the purposes of reporting domain and theme totals below, these articles have been counted as one in order to avoid artificially inflating their prevalence, though questionnaire response tallies have been reported separately where relevant. The questionnaire used by Cohen et al. [27] was not available in its entirety online, so only items included in the paper body itself could be included for analysis.

In total, 75 unique items were identified across the 7 studies, comprising 71 questionnaire items and 4 participant quotes (See Table, Supplementary Digital Content 5 for full dataset). At this stage, some additional items were removed via consensus between authors, as they asked about current behaviour only, rather than potential barriers and enablers; e.g., “Do you persuade your elderly patients to try a [Hearing Aid]?”.

Ten of the fourteen domains of the TDF were represented by at least one item across the 7 studies (Table 2). Four domains did not occur on any questionnaire tool: Behavioural regulation, Emotion, Reinforcement, and Social/Professional role and identity. The number of themes identified under each represented domain ranged from one to five, and loosely associated with the number of individual items in each domain. Knowledge and Beliefs about consequences were the most frequently included domains (n = 28 and n = 18, respectively), while Goals occurred across the highest number of studies (n = 5 out of a possible 6). Table 2 shows the number of items per domain across all reviewed studies.

**Table 2:** Number of items (i.e. quote or questionnaire item) per domain across studies

	Danhauer et al. 2008 [28], Johnson et al. 2008 [29]	Sydlowski et al. 2022 [32]	Gilliver and Hickson, 2011 [30]	Zazove et al. 2017 [31]	Parving et al. 1996 [26]	Cohen et al. 2005 [27]	Totals per domain
Knowledge	6	15	6	1			28
Beliefs about consequences	2		8		8		18
Social influences		2	4			2	8
Goals (priorities)	2	1	2	1		1	7
Beliefs about capabilities			2	2			4
Memory, attention and decision processes						4	4
Environmental context	1					2	3
Skills	1						1
Optimism			1				1
Intentions	1						1
Total items:	13	18	23	4	8	9	75
Total domains:	6	3	6	3	1	4	

All themes are described narratively below, regardless of the number of studies they appeared in, in the order shown in Table 2. A summary of whether these themes were expressed as potential barriers or enablers can be seen in Table 3.

**Table 3:** Summary of barriers and enablers identified across all articles

Citation	Country	Barriers identified	Enablers identified
Parving et al. [26]	Denmark	Nil	Positive beliefs regarding the social outcomes of hearing care Positive beliefs about hearing rehab outcomes/efficacy
Cohen et al. [27]	USA	Lack of time Lack of resources Higher relative importance of other health conditions	Someone initiates the discussion about hearing
Danhauer et al. [28]^b^	USA	Lack of familiarity with diagnostic tools and criteria Low knowledge of treatments Lack of intention to change future behaviour Lack of time	High knowledge of impacts and comorbidities High knowledge of prevalence Positive beliefs regarding the skills of other health professionals Positive beliefs about hearing rehab outcomes/efficacy Lower or equal relative importance of other health conditions Someone initiates the discussion about hearing
Johnson et al. [29]^b^	USA	Lack of familiarity with diagnostic tools and criteria Low knowledge of treatments Negative beliefs about hearing rehab outcomes/efficacy^a^ Lack of intention to change future behaviour Lack of time	High knowledge of impacts and comorbidities High knowledge of prevalence Positive beliefs regarding the skills of other health professionals Positive beliefs about hearing rehab outcomes/efficacy^a^ Lower or equal relative importance of other health conditions Someone initiates the discussion about hearing
Gilliver & Hickson [30]	Australia	Low knowledge of prevalence Unrealistic Optimism Negative beliefs about patient uptake of hearing care Higher relative importance of other health conditions^a^ Perceived Social Stigma	High knowledge of impacts and comorbidities High knowledge of treatments High knowledge of prevalence Ease of discussion Familiarity with next steps Positive beliefs about hearing rehab outcomes/efficacy Lower or equal relative importance of other health conditions^a^
Zazove et al. [31]	USA	Low knowledge of treatments Low ease of discussion Lack of familiarity with next steps Higher relative importance of other health conditions	Nil
Sydlowski et al. [32]^c^	USA	Lack of familiarity with diagnostic tools and criteria^a^ Low knowledge of impacts and comorbidities^a^ Higher relative importance of other health conditions	Familiarity with diagnostic tools and criteria^a^ High knowledge of impacts and comorbidities^a^

^a^Indicates a theme that was identified as both a barrier and enabler in a given study

^b^Indicates studies that used the same questionnaire tool

^c^Indicates complete results were not reported in this paper—e.g., only the percentage of “strongly agree” responses were reported. Where the reported results did not constitute a majority, they are not reported as a clear barrier or an enabler

### Knowledge

*Theme: Familiarity with diagnostic criteria and tools (Three studies)*

This theme explored the familiarity of GPs with the diagnostic criteria for hearing loss, as well as tools for assessing hearing and hearing handicap.

In all cases, familiarity with diagnostic criteria and tools was classified as a barrier; GPs reported that their knowledge of diagnostic criteria and tools was low, and that more information on this topic would be welcomed [28, 29, 32].

*Theme: Knowledge of Impacts and comorbidities (Four studies)*

GPs’ knowledge of medical comorbidities associated with hearing loss was generally lower than their knowledge of the impacts of hearing loss on social connectedness and independence. Across studies, awareness of the link between hearing loss and specific medical conditions ranged from 51% for depression, to 9% for type 2 diabetes, while 77% to 99% of respondents agreed that hearing loss impacted quality of life and social outcomes [28–30, 32].

*Theme: Knowledge of treatments (Five studies)*

*“There’s no good answer for what to do if [patients] have a hearing loss.”* (Zazove et al. [31], p 277)

Across studies, there was a general lack of awareness of treatments for hearing loss among responding GPs [28–32]. Questionnaire data from Sydlowski et al. [32] found only just over one third of respondents strongly agreed that hearing loss was treatable. Similarly, Danhauer et al. [28] and Johnson et al. [29] reported significant variation among respondents regarding whether medical treatments (as opposed to audiologist-driven hearing care) were appropriate for hearing loss, with 36.7–60.7% of participants indicating they were unsure of the proportion of “hearing loss that [could be] treated medically”.

*Theme: Knowledge of Prevalence (Four studies)*

Awareness of the prevalence of hearing loss was relatively consistent, with respondents generally agreeing that between 33 and 46% of older patients were likely to have a hearing loss [28–30].

Hearing loss normalisation among older patients was also common, with 43% of respondents to Gilliver & Hickson [30] agreeing that “almost all of my older patients have hearing difficulties”, and 20% of respondents in the study by Sydlowski et al. [32] strongly agreeing that “hearing loss is a normal part of ageing”.

*Theme: Knowledge of condition (aetiology, symptoms etc.) (One study)*

GP knowledge of the causes and symptoms of hearing loss emerged as a theme in one study only [32]. Only one item was presented: “Hearing loss is preventable”, with which 19% strongly agreed. While this does not directly relate to hearing care and outcomes, it is possible that this relates to perceptions of hearing loss prevalence, where hearing loss is viewed as an unpreventable facet of ageing. Overall, self-identified knowledge of hearing loss as a medical condition was mixed across all themes and studies.

### Beliefs about consequences

*Theme: Beliefs about hearing care outcomes/efficacy (Four studies)*

Most GPs held positive beliefs regarding hearing care outcomes, with high levels of agreement that hearing aids were effective, that the pros of hearing aids outweighed the cons, and that older patients typically achieved good outcomes with hearing aids [26, 28–30].

*Theme: Social outcomes of hearing care (One study)*

GPs were generally positive when asked if hearing aids could fully or partially remediate a hearing problem across a variety of social settings, with the highest agreement for face-to-face conversation (100%), and the lowest for listening at a party (55%) [26].

*Theme: Beliefs about patient uptake of hearing care (One study)*

This theme explored GPs’ beliefs on how readily their patients would take to hearing care. Gilliver & Hickson [30] found that 76% of respondents agreed that “many older patients find it difficult to adjust to using hearing aids”, and similarly found that less than half of respondents agreed that “older patients will generally take/follow up the hearing rehabilitation advice given to them”.

Overall, practitioner views on the outcomes of hearing care amongst those who receive it were generally positive. However, variation existed in beliefs of how readily patients would adapt to hearing care, and whether they would act on hearing advice.

### Social influences

*Theme: Someone initiates the discussion about hearing (Two studies)*

GPs typically reported relying on the patient to bring up hearing as a discussion topic, rather than broaching it themselves. Of the respondents who reported never, or only occasionally, assessing for hearing loss in the study by Cohen et al. [27], 17.6% reported that they would evaluate hearing loss if the patient reported a problem. Sydlowski et al. [32] included two items under this theme: “What percent of your patients 50 years of age or older proactively ask you themselves or have a loved one ask you about their hearing health?” and “What percent of your patients 50 years of age or older wait for you to initiate conversations about their hearing health?”; however the responses to these questions were not reported. Overall, most practitioners agreed that a patient report of hearing difficulty would warrant on-referral to a hearing professional, however whether GPs feel they should wait until this is flagged by the patient, or raise it proactively was unclear.

*Theme: Social stigma (One study)*

The perceived social stigma of hearing aids was raised as a factor which GPs felt affected the ease with which they could raise the topic of hearing [30]. GPs generally agreed that their patients were concerned about the stigma of wearing hearing aids, and that they would prefer to hide or deny their hearing loss. Overall, the perceived stigma of hearing loss/hearing aids was high among the surveyed practitioners.

### Goals

*Theme: The relative importance of other health conditions (Six studies)*

The relative importance of hearing loss when compared to other health conditions in older patients was a frequently occurring theme across studies. However, there was significant variation in how important respondents felt hearing loss was across studies, with participants in three studies rating hearing loss as a low priority health condition [27, 31, 32], while the remaining three studies identified hearing loss as a high priority amongst GPs [28–30].

### Beliefs about capabilities

*Theme: Ease of discussion (Two studies)*

*“I don’t have a script to address patients with hearing loss.”* (Zazove et al. [31], p 277)

Items which fell under this theme explored GP beliefs about the ease with which they are able to discuss hearing loss with their patients. This theme was raised as both a barrier and enabler, with interviews by Zazove et al. [31] indicating a lack of confidence in conducting these discussions, while questionnaire data from Gilliver & Hickson [30] found that over half of all participants found it “easy to discuss hearing rehabilitation and assessment with older patients”.

*Theme: Familiarity with next steps (Two studies)*

*“I feel unprepared to answer patient’s questions about hearing loss and treatment options.”* (Zazove et al. [31], p 277)

This theme related to GP confidence in explaining and carrying out the steps in the hearing care referral pathway. Qualitative data, such as that quoted above, indicated lower levels of confidence than did the quantitative data: of the 23 interviews conducted by Zazove et al. [31], 12 included reference to the “mental model of [hearing loss]” (p 278) or their overall familiarity and confidence in thinking about or discussing hearing loss as being a barrier to following an electronic prompt to refer patients to audiology. This included content relating to GP familiarity with next steps, however the exact number of interviews in which this theme arose is unknown. However, familiarity with next steps was classified as an enabler in the quantitative data, with nearly two thirds of respondents to Gilliver and Hickson [30] agreeing that “it is easy/straightforward to refer older patients for hearing assessment/rehabilitation”.

Overall, responses to “beliefs about capabilities” items were mixed, with variation observed across studies. Questionnaire responses tended to indicate more positive perceptions of this domain than did qualitative responses; though the small number of qualitative items available makes the importance of this difference difficult to interpret.

### Memory, attention and decision processes

*Theme: Abnormal test results as a trigger for referral (One study)*

The use of abnormal test results as the trigger for referring to a hearing health professional arose as a theme in one study [27]. Respondents identified the following factors as indications for referral: abnormal audiogram (29.4%), abnormal health questionnaire (20%), abnormal tympanometry (17.6%), and abnormal tuning fork test (15.3%).

### Environmental context and resources

*Theme: Lack of time (Three studies)*

Over half of the respondents to Danhauer et al. [28] and Johnson et al. [29] agreed with the statement “I do not have time to do routine hearing and/or balance screenings for my elderly patients” at 56.7% and 54.6%, respectively. Further, 38.2% of respondents to Cohen et al. [27] identified “not enough time” as a factor for why hearing loss was not explored; sharing equal highest prevalence with “more pressing issues”.

*Theme: Lack of resources (One study)*

A lack of resources in the local area arose as a theme in one study [27]. When asked why hearing loss was not evaluated in appointments with older patients, 8.8% of respondents identified “No local otolaryngologist/audiologist” as a factor.

### Optimism

*Theme: Unrealistic optimism (One study)*

The theme of “unrealistic optimism” (that is, optimism that positive outcomes will be achieved even without intervention) was identified in one study [30]. A total of 60% of respondents agreed with the statement “Hearing deteriorates with age, and does not always require amplification”, with 18% disagreeing and 21% responding neutrally.

### Intentions

*Theme: Intention to change future behaviour (Two studies)*

Both Danhauer et al. [28] and Johnson et al. [29] posed the question “If you did not use the HHIE…previously, will you use them now to screen your elderly patients?”. The Hearing Handicap Inventory (HHIE) was designed to assess self-perceived hearing handicap in older adults. Despite both studies utilising the same questionnaire, response patterns differed; 28.4% of respondents to Johnson et al. [29] indicated they would make use of the HHIE in future, while only 7.4% of respondents to Danhauer et al. [28] answered affirmatively. Around one third of respondents to both questionnaires answered “no” to this question, while the largest group (59.3% in Danhauer et al. [28] and 43.2% in Johnson et al. [29]) responded that they were unsure.

### Skills

*Theme: Skills of other health professionals (Two studies)*

The concept of whether other health professionals in the referral pathways possessed the relevant skills was identified in both Danhauer et al. [28] and Johnson et al. [29], In both cases, over half of the sample (56.7% and 51.7%, respectively) agreed that “Audiologists are well qualified to diagnose and manage non-medical hearing and balance problems in the elderly”. Around one third (33.3% and 34.5%, respectively) responded neutrally to this question.

## Discussion

This systematic review aimed to identify the potential barriers and enablers which underpin the low rates of GP referral to audiology and to synthesise them using the Theoretical Domains Framework.

Nine themes could be definitively classified, as they occurred in more than one study, and in the majority of occurrences there was agreement on whether they constituted a barrier or an enabler. Four main potential barriers were identified in this review: “Lack of time”, “Familiarity with diagnostic criteria and tools”, “Knowledge of treatments” and “Relative importance of other health conditions”. A further five potential enablers were also identified: “Knowledge of impacts and comorbidities”, “Knowledge of prevalence”, “Beliefs about hearing care outcomes/efficacy”, “Someone initiates the discussion about hearing” and “Skills of other health professionals”.

The majority of studies identified in this review focused on the domains of “Knowledge” and “Beliefs about consequences”, with over half of all items falling within these domains. It is therefore unsurprising that the majority of barriers and enablers identified in this review fall under these two domains. Therefore, some caution is needed in interpreting these findings as being representative of the relative importance of these two domains.

Overall, there was a paucity of data in this area, and findings across the included studies were mixed and in some cases contradictory. Of the themes that could be classified into potential “barriers” or “enablers”, only one potential barrier (“Lack of time”) and two potential enablers (“Someone initiates the discussion about hearing loss” and “Skills of other health professionals”) did not appear in both categories across separate studies. No single study encompassed all 10 of the identified domains, with the number of domains represented in each article ranging from 1 to 6. While not all domains were expected to be relevant in every setting, overlap in thematic content between articles was low (Table 2). This suggests that each study offered only a small window of insight into the potential barriers and enablers to GP referral for hearing care, and that data saturation in this area has not yet been reached. Therefore, further exploration of this topic is needed to fully understand the factors driving GP referral behaviours.

Interestingly, the two questionnaire tools that addressed the greatest number of domains were that used by Johnson et al. [29] and Danhauer et al. [28]; and that used by Gilliver and Hickson [30]. These were the only tools to be designed using stakeholder (GP) input and a theoretical framework, respectively. Studies that scored lower on the QuADS criterion “*Theoretical or conceptual underpinning to the research”* tended to cover fewer domains than those that scored highly. The exception to this was Zazove et al. [31], which was rated highly on this criterion and yet addressed only 3 domains (Knowledge, Beliefs about Capabilities and Goals). However, this may be due to the low number of items included in total, with only four direct participant quotes available for coding. While only one of the questionnaire tools assessed in this review explicitly incorporated a stakeholder voice into its design, it is possible that other questionnaires were developed with GP input and authors simply did not report this. Therefore, caution should be taken in interpreting items derived from stakeholder-informed articles as having greater validity than those from other articles included in this review.

### Limitations and strengths

The majority of studies included in this review were conducted in the USA, which may be a consequence of including only studies available in English for data extraction. As a result, the findings of this review may not be applicable to other health jurisdictions. The small number of studies in this area and the general lack of consensus between studies limited the interpretation of study findings. A further limitation of this review is that it did not include grey literature. It should also be noted that many of the studies included in this review were not designed to investigate barriers and enablers directly, and this may have influenced how the data was presented in the primary studies, as well as its interpretation. While efforts were made to code all relevant data into the TDF, the use of deductive coding prior to inductive coding may have created a risk of missing data which did not ‘fit’ into this framework.

To the best of our knowledge, this is the first systematic review to investigate potential barriers and enablers to GP referral for hearing care in older adults. Including both qualitative and quantitative literature facilitated a comprehensive overview of the published literature to date. A recent systematic review exploring patient factors which influence the uptake of hearing devices identified 42 relevant studies, highlighting the comparatively small body of literature exploring GP related factors [33]. While there has been minimal behaviour change research on this topic, implementation research has frequently been carried out in general practice, and the theory-informed identification of potential barriers and enablers is an important step in this process [34–37]. The 10 domains identified in this review represent a far greater thematic scope than has previously been explored in any study on this topic to date.

### Implications for future research

Evidence-practice gaps occur frequently across a range of clinical fields [38]. Between 50 and 80% of proposed evidence-based practices never make it to clinical practice, and for those that do, the median reported delay to becoming part of routine clinical care is 17 years [39]. Evidence-practice gaps can therefore not be addressed by passive approaches such as demonstrating that a practice is evidence-based, and then waiting for it to trickle into clinical practice. Active approaches to implementation of such evidence into clinical practice are required to improve patient care in a timely and equitable manner.

Given the low proportion of qualitative studies identified in this review, adding to the body of qualitative research is a high priority for future exploration of this topic. Future research may assist in clarifying which barriers and enablers are the highest priority targets for future interventions to improve GP referrals of older adults to hearing care, helping bridge the evidence-practice gap in this field.

## Conclusions

In conclusion, this review identified four potential barrier themes and five potential enabler themes in the thematic content of the published literature. However, the low number of studies in this area, and the overall lack of consensus between studies makes interpolation of these findings difficult. In addition, the lack of agreement between studies may suggest that data saturation has not been reached, and further research is needed to further develop our understanding of the barriers and enablers to GP referral to hearing care. Future research would benefit from explicitly involving GPs in the design of data collection tools, and by making use of qualitative data collection methods where possible. This may allow for a greater number of emergent themes to be discovered than would be possible through use of the existing questionnaire tools described in this review.

## Supplementary Information

Below is the link to the electronic supplementary material.

Supplementary file1 (DOCX 46 KB)

## Data Availability

Most of the data supporting this review are available within the article and its supplementary information, further information is available on request from the authors.
