# Supplementary material for: Barriers and enablers to general practitioner referral of older adults to hearing care: a systematic review using the theoretical domains framework
Source: Eur Geriatr Med. 2025 May 22;16(4):1507–18. doi: 10.1007/s41999-024-01124-5 (PMC12378124; doi:10.1007/s41999-024-01124-5)
Supplement: Supplementary file 1 — Supplementary file1 (DOCX 46 KB) [file 41999_2024_1124_MOESM1_ESM.docx]

**Supplementary Information**

**Title:**

Barriers and Enablers to General Practitioner Referral of Older Adults to Hearing Care: A Systematic Review Using the Theoretical Domains Framework

**Journal:**

European Geriatric Medicine

**Authors:**

E C Davine^1^, P A Busby^1^, S Peters^1^, J J Francis ^1, 2^, J Z Sarant ^1^

^1^ The University of Melbourne

^2^ Ottawa Hospital Research Institute

**Corresponding Author:**

Ella Davine, 550 Swanston Street, Carlton, VIC 3053, Australia. Phone: +61 8344 4213, Email: [e.davine@unimelb.edu.au](mailto:e.davine@unimelb.edu.au)

**Online resource 1:** The 14 domains of the TDF (from Atkins et al. [20] reproduced under Creative Commons Licence.)

| **Domain** | **Definition** |
| --- | --- |
| Knowledge | An awareness of the existence of something |
| Skills | An ability or proficiency acquired through practice |
| Social/professional role and identity | A coherent set of behaviours and displayed personal qualities of an individual in a social or work setting |
| Beliefs about capabilities | Acceptance of the truth, reality or validity about an ability, talent, or facility that a person can put to constructive use |
| Optimism | The confidence that things will happen for the best or that desired goals will be attained |
| Beliefs about consequences | Acceptance of the truth, reality, or validity about outcomes of a behaviour in a given situation |
| Reinforcement | Increasing the probability of a response by arranging a dependent relationship, or contingency, between the response and a given stimulus |
| Intentions | A conscious decision to perform a behaviour or a resolve to act in a certain way |
| Goals | Mental representations of outcomes or end states that an individual wants to achieve |
| Memory, attention and decision processes | The ability to retain information, focus selectively on aspects of the environment and choose between two or more alternatives |
| Environmental context and resources | Any circumstance of a person’s situation or environment that discourages or encourages the development of skills and abilities, independence, social competence, and adaptive behaviour |
| Social influences | Those interpersonal processes that can cause individuals to change their thoughts, feelings, or behaviours |
| Emotion | A complex reaction pattern, involving experiential, behavioural, and physiological elements, by which the individual attempts to deal with a personally significant matter or event |
| Behavioural regulation | Anything aimed at managing or changing objectively observed or measured actions |

**Online Resource 2:** Preferred Reporting Items for Systematic Reviews and Meta-Analyses (PRISMA) Checklist

| **Section and Topic** | **Item #** | **Checklist item** | **Location where item is reported** |
| --- | --- | --- | --- |
| **TITLE** | | |  |
| Title | 1 | Identify the report as a systematic review. | Line 3 |
| **ABSTRACT** | | |  |
| Abstract | 2 | See the PRISMA 2020 for Abstracts checklist. | Line 21-42 |
| **INTRODUCTION** | | |  |
| Rationale | 3 | Describe the rationale for the review in the context of existing knowledge. | Line 46-71 |
| Objectives | 4 | Provide an explicit statement of the objective(s) or question(s) the review addresses. | Line 87-90 |
| **METHODS** | | |  |
| Eligibility criteria | 5 | Specify the inclusion and exclusion criteria for the review and how studies were grouped for the syntheses. | Line 104-126 |
| Information sources | 6 | Specify all databases, registers, websites, organisations, reference lists and other sources searched or consulted to identify studies. Specify the date when each source was last searched or consulted. | Line 96-98 |
| Search strategy | 7 | Present the full search strategies for all databases, registers and websites, including any filters and limits used. | SDC 3 |
| Selection process | 8 | Specify the methods used to decide whether a study met the inclusion criteria of the review, including how many reviewers screened each record and each report retrieved, whether they worked independently, and if applicable, details of automation tools used in the process. | Line 128-132 |
| Data collection process | 9 | Specify the methods used to collect data from reports, including how many reviewers collected data from each report, whether they worked independently, any processes for obtaining or confirming data from study investigators, and if applicable, details of automation tools used in the process. | Line 146-152 |
| Data items | 10a | List and define all outcomes for which data were sought. Specify whether all results that were compatible with each outcome domain in each study were sought (e.g. for all measures, time points, analyses), and if not, the methods used to decide which results to collect. | Line 149-165, Figure 1 |
|  | 10b | List and define all other variables for which data were sought (e.g. participant and intervention characteristics, funding sources). Describe any assumptions made about any missing or unclear information. | N/A |
| Study risk of bias assessment | 11 | Specify the methods used to assess risk of bias in the included studies, including details of the tool(s) used, how many reviewers assessed each study and whether they worked independently, and if applicable, details of automation tools used in the process. | Line 134-144 |
| Effect measures | 12 | Specify for each outcome the effect measure(s) (e.g. risk ratio, mean difference) used in the synthesis or presentation of results. | N/A |
| Synthesis methods | 13a | Describe the processes used to decide which studies were eligible for each synthesis (e.g. tabulating the study intervention characteristics and comparing against the planned groups for each synthesis (item #5)). | N/A |
|  | 13b | Describe any methods required to prepare the data for presentation or synthesis, such as handling of missing summary statistics, or data conversions. | Line 154-165 |
|  | 13c | Describe any methods used to tabulate or visually display results of individual studies and syntheses. | N/A |
|  | 13d | Describe any methods used to synthesize results and provide a rationale for the choice(s). If meta-analysis was performed, describe the model(s), method(s) to identify the presence and extent of statistical heterogeneity, and software package(s) used. | Line 154-165 |
|  | 13e | Describe any methods used to explore possible causes of heterogeneity among study results (e.g. subgroup analysis, meta-regression). | N/A |
|  | 13f | Describe any sensitivity analyses conducted to assess robustness of the synthesized results. | N/A |
| Reporting bias assessment | 14 | Describe any methods used to assess risk of bias due to missing results in a synthesis (arising from reporting biases). | N/A |
| Certainty assessment | 15 | Describe any methods used to assess certainty (or confidence) in the body of evidence for an outcome. | N/A |
| **RESULTS** | | |  |
| Study selection | 16a | Describe the results of the search and selection process, from the number of records identified in the search to the number of studies included in the review, ideally using a flow diagram. | Line 192-197, figure 2 |
|  | 16b | Cite studies that might appear to meet the inclusion criteria, but which were excluded, and explain why they were excluded. | N/A |
| Study characteristics | 17 | Cite each included study and present its characteristics. | Line 200-201, Table 1 |
| Risk of bias in studies | 18 | Present assessments of risk of bias for each included study. | SDC 4 |
| Results of individual studies | 19 | For all outcomes, present, for each study: (a) summary statistics for each group (where appropriate) and (b) an effect estimate and its precision (e.g. confidence/credible interval), ideally using structured tables or plots. | N/A |
| Results of syntheses | 20a | For each synthesis, briefly summarise the characteristics and risk of bias among contributing studies. | N/A |
|  | 20b | Present results of all statistical syntheses conducted. If meta-analysis was done, present for each the summary estimate and its precision (e.g. confidence/credible interval) and measures of statistical heterogeneity. If comparing groups, describe the direction of the effect. | N/A |
|  | 20c | Present results of all investigations of possible causes of heterogeneity among study results. | N/A |
|  | 20d | Present results of all sensitivity analyses conducted to assess the robustness of the synthesized results. | N/A |
| Reporting biases | 21 | Present assessments of risk of bias due to missing results (arising from reporting biases) for each synthesis assessed. | N/A |
| Certainty of evidence | 22 | Present assessments of certainty (or confidence) in the body of evidence for each outcome assessed. | N/A |
| **DISCUSSION** | | |  |
| Discussion | 23a | Provide a general interpretation of the results in the context of other evidence. | Line 402-409 |
|  | 23b | Discuss any limitations of the evidence included in the review. | Line 442-452 |
|  | 23c | Discuss any limitations of the review processes used. | Line 442-452 |
|  | 23d | Discuss implications of the results for practice, policy, and future research. | Line 466-477 |
| **OTHER INFORMATION** | | |  |
| Registration and protocol | 24a | Provide registration information for the review, including register name and registration number, or state that the review was not registered. | Line 94-95 |
|  | 24b | Indicate where the review protocol can be accessed, or state that a protocol was not prepared. | Line 94-95 |
|  | 24c | Describe and explain any amendments to information provided at registration or in the protocol. | N/A |
| Support | 25 | Describe sources of financial or non-financial support for the review, and the role of the funders or sponsors in the review. | Title Page |
| Competing interests | 26 | Declare any competing interests of review authors. | Title Page |
| Availability of data, code and other materials | 27 | Report which of the following are publicly available and where they can be found: template data collection forms; data extracted from included studies; data used for all analyses; analytic code; any other materials used in the review. | SDC 5 |

*From:*  Page MJ, McKenzie JE, Bossuyt PM, Boutron I, Hoffmann TC, Mulrow CD, et al. The PRISMA 2020 statement: an updated guideline for reporting systematic reviews. BMJ 2021;372:n71. doi: 10.1136/bmj.n71

**Online Resource 3:** Full search strategies for each database

| **Database** | **Search Strategy** | **Number of results** |
| --- | --- | --- |
| **Ovid*** | (GP* OR General Prac* OR Primary *care OR PCP OR family doctor* OR family physician* OR family medicine OR medical practitioner* OR Primary Health* Provider*) AND Refer* AND (Hearing care OR Audiolog* OR hearing aid* OR hearing loss* OR hearing impair*) AND (Adult* OR (over ADJ4 60) OR (over ADJ4 65) OR (over ADJ4 50) OR (over ADJ4 55) OR older adult* OR Elder* OR Ageing OR geriatric*) | **136** |
| **Scopus*** | (GP* OR General Prac* OR Primary *care OR PCP OR family doctor* OR family physician* OR family medicine OR medical practitioner* OR Primary Health* Provider*) AND Refer* AND (Hearing care OR Audiolog* OR hearing aid* OR hearing loss* OR hearing impair*) AND (Adult* OR (over ADJ4 60) OR (over ADJ4 65) OR (over ADJ4 50) OR (over ADJ4 55) OR older adult* OR Elder* OR Ageing OR geriatric*) | **574** |
| **CINAHL**** | GP* OR General Prac* OR Primary care* OR PCP OR family doctor* OR family physician* OR family medicine OR medical practitioner* OR Primary Health* Provider* OR (MH "Physicians, family") AND Refer* OR (MH "Referral and Consultation") AND Hearing care OR Audiolog* OR hearing aid* OR hearing loss* OR hearing impair* OR (MH “Hearing Disorders”) OR (MH “Presbycusis”) AND Adult* OR over 60 OR older adult* OR Elder* OR Ageing OR geriatric* OR (over ADJ4 60) OR (over ADJ4 65) OR (over ADJ4 50) OR (over ADJ4 55) OR (MH "Aged") OR (MH "Aged, 80 and Over") | **87** |

***** = Search strategies used were identical, but subject header systems within each database utilised independently.

****** = Search terms and subject headers integrated into a single search strategy.

**Online Resource 4:** Quality assessment using the QuADS. A score of 0 indicates the criteria was not addressed, a score of 3 indicates the criteria was comprehensively met.

| **QuADS criterion** | **Paper** | | | | | | |
| --- | --- | --- | --- | --- | --- | --- | --- |
|  | **Cohen et al., 2005** | **Danhauer et al., 2008** | **Gilliver & Hickson, 2011** | **Johnson et al., 2008** | **Parving et al., 1996** | **Sydlowski et al., 2022** | **Zazove et al., 2017** |
| **1. Theoretical or conceptual underpinning to the research** | **1**  *(General reference to broad theories or concepts that frame the study)* | **3**  *(Explicit discussion of the theories or concepts that inform the study, with application of the theory or concept evident through the design, materials and outcomes explored)* | **3**  *(Explicit discussion of the theories or concepts that inform the study, with application of the theory or concept evident through the design, materials and outcomes explored)* | **3**  *(Explicit discussion of the theories or concepts that inform the study, with application of the theory or concept evident through the design, materials and outcomes explored)* | **1**  *(General reference to broad theories or concepts that frame the study)* | **2**  *(Identification of specific theories or concepts that frame the study and how these informed the work undertaken)* | **3**  *(Explicit discussion of the theories or concepts that inform the study, with application of the theory or concept evident through the design, materials and outcomes explored)* |
| **2. Statement of research aim/s** | **1**  *(Reference to what the sought to achieve embedded within the report but no explicit aims statement.)* | **3**  *(Explicit and detailed statement of aim/s in the main body of report)* | **3**  *(Explicit and detailed statement of aim/s in the main body of report)* | **3**  *(Explicit and detailed statement of aim/s in the main body of report)* | **2**  *(Aims statement made but may only appear in the abstract or be lacking detail)* | **2**  *(Aims statement made but may only appear in the abstract or be lacking detail)* | **1**  *(Reference to what the sought to achieve embedded within the report but no explicit aims statement.)* |
| **3. Clear description of research setting and target population** | **3**  *(Specific description of the research setting and target population of study)* | **3**  *(Specific description of the research setting and target population of study)* | **3**  *(Specific description of the research setting and target population of study)* | **3**  *(Specific description of the research setting and target population of study)* | **3**  *(Specific description of the research setting and target population of study)* | **2**  *(Description of research setting is made but is lacking detail)* | **2**  *(Description of research setting is made but is lacking detail)* |
| **4. The study design is appropriate to address the stated research aim/s** | **2**  *(The study design can address the stated research aim/s but there is a more suitable alternative that could have been used or used in addition)* | **2**  *(The study design can address the stated research aim/s but there is a more suitable alternative that could have been used or used in addition)* | **3**  *(The study design selected appears to be the most suitable approach to attempt to answer the stated research aim/s)* | **3**  *(The study design selected appears to be the most suitable approach to attempt to answer the stated research aim/s)* | **1**  *(The study design can only address some aspects of the stated research aim/s)* | ***1***  *(The study design can only address some aspects of the stated research aim/s)* | **3**  *(The study design selected appears to be the most suitable approach to attempt to answer the stated research aim/s)* |
| **5. Appropriate sampling to address the research aim/s** | **0**  *(No mention of the sampling approach.)* | **3**  *(Detailed evidence of consideration of the sample required to address the research aim/s)* | **0**  *(No mention of the sampling approach.)* | **2**  *(Evidence of consideration of sample required to address the aim)* | **2**  *(Evidence of consideration of sample required to address the aim)* | **1**  *(Evidence of consideration of the sample required)* | **0**  *(No mention of the sampling approach)* |
| **6. Rationale for choice of data collection tool/s** | **0**  *(No mention of rationale for data collection tool used)* | **3**  *(Detailed explanation of rationale for choice of data collection tool/s)* | **3**  *(Detailed explanation of rationale for choice of data collection tool/s)* | **2**  *(Basic explanation of rationale for choice of data collection tool/s)* | **0**  *(No mention of rationale for data collection tool used)* | **0**  *(No mention of rationale for data collection tool used)* | **2**  *(Basic explanation of rationale for choice of data collection tool/s)* |
| **7. The format and content of data collection tool is appropriate to address the stated research aim/s** | **1**  *(Structure and/or content of tool/s suitable to address some aspects of the research aim/s or to address the aim/s superficially)* | **3**  *(Structure and content of tool/s allow for detailed data to be gathered around all relevant issues required to address the stated research aim/s)* | **3**  *(Structure and content of tool/s allow for detailed data to be gathered around all relevant issues required to address the stated research aim/s)* | **2**  *(Structure and/or content of tool/s allow for data to be gathered broadly addressing the stated aim/s but could benefit from refinement)* | **1**  *(Structure and/or content of tool/s suitable to address some aspects of the research aim/s or to address the aim/s superficially)* | **1**  *(Structure and/or content of tool/s suitable to address some aspects of the research aim/s or to address the aim/s superficially)* | **2**  *(Structure and/or content of tool/s allow for data to be gathered broadly addressing the stated aim/s but could benefit from refinement)* |
| **8. Description of data collection procedure** | **3**  *(Detailed description of each stage of the data collection procedure, including when, where and how data was gathered such that the procedure could be replicated)* | **3**  *(Detailed description of each stage of the data collection procedure, including when, where and how data was gathered such that the procedure could be replicated)* | **3**  *(Detailed description of each stage of the data collection procedure, including when, where and how data was gathered such that the procedure could be replicated)* | **3**  *(Detailed description of each stage of the data collection procedure, including when, where and how data was gathered such that the procedure could be replicated)* | **1**  *(Basic and brief outline of data collection procedure)* | **1**  *(Basic and brief outline of data collection procedure)* | **3**  *(Detailed description of each stage of the data collection procedure, including when, where and how data was gathered such that the procedure could be replicated)* |
| **9. Recruitment data provided** | **3**  *(Complete data allowing for full picture of recruitment outcomes)* | **3**  *(Complete data allowing for full picture of recruitment outcomes)* | **1**  *(Minimal and basic recruitment data)* | **3**  *(Complete data allowing for full picture of recruitment outcomes)* | **2**  *(Some recruitment data but not a complete account)* | **2**  *(Some recruitment data but not a complete account)* | **1**  *(Minimal and basic recruitment data)* |
| **10. Justification for analytic method selected** | **2**  *(Basic justification for choice of analytic method selected)* | **1**  *(Very limited justification for choice of analytic method selected)* | **1**  *(Very limited justification for choice of analytic method selected)* | **2**  *(Basic justification for choice of analytic method selected)* | **1**  *(Very limited justification for choice of analytic method selected)* | **2**  *(Basic justification for choice of analytic method selected)* | **2**  *(Basic justification for choice of analytic method selected)* |
| **11. The method of analysis was appropriate to answer the research aim/s** | **2**  *(Method of analysis can address the research aim/s but there is a more suitable alternative that could have been used or used in addition to offer a stronger analysis)* | **1**  *(Method of analysis can only address the research aim/s basically or broadly.)* | **1**  *(Method of analysis can only address the research aim/s basically or broadly.)* | **2**  *(Method of analysis can address the research aim/s but there is a more suitable alternative that could have been used or used in addition to offer a stronger analysis)* | **1**  *(Method of analysis can only address the research aim/s basically or broadly.)* | **2**  *(Method of analysis can address the research aim/s but there is a more suitable alternative that could have been used or used in addition to offer a stronger analysis)* | **2**  *(Method of analysis can address the research aim/s but there is a more suitable alternative that could have been used or used in addition to offer a stronger analysis)* |
| **12. Evidence that the research stakeholders have been considered in research design or conduct.** | **0**  *(No mention at all.)* | **2**  *(Evidence of stakeholder input informing the research)* | **0**  *(No mention at all.)* | **2**  *(Evidence of stakeholder input informing the research)* | **0**  *(No mention at all.)* | **0**  *(No mention at all.)* | **0**  *(No mention at all.)* |
| **13. Strengths and limitations critically discussed** | **2**  *(Discussion of some of the key strengths and weaknesses of the study but not complete)* | **3**  *(Thorough discussion of strengths and limitations of all aspects of study including design, methods, data collection tools, sample & analytic approach)* | **2**  *(Discussion of some of the key strengths and weaknesses of the study but not complete)* | **3**  *(Thorough discussion of strengths and limitations of all aspects of study including design, methods, data collection tools, sample & analytic approach)* | **0**  *(No mention at all.)* | **2**  *(Discussion of some of the key strengths and weaknesses of the study but not complete)* | **1**  *(Very limited mention of strengths and limitations with omissions of many key issues)* |

**Online Resource 5:** Domain and theme breakdown for all items

| **Question** | **Domain** | **Theme** | **Paper** | **Location in text** |
| --- | --- | --- | --- | --- |
| Were you aware of the attached screening instruments (HHIE and DHI) before receiving them with this survey? | Knowledge | Familiarity with diagnostic criteria and tools | Danhauer et al. 2008, Johnson et al. 2008 | Table 1. item 9 |
| the HHIE an effective self-report hearing screening method for the elderly? | Knowledge | Familiarity with diagnostic criteria and tools | Danhauer et al. 2008, Johnson et al. 2008 | Table 2. item 15 |
| Would you like additional information about hearing and balance screening in the elderly? | Knowledge | Familiarity with diagnostic criteria and tools | Danhauer et al. 2008, Johnson et al. 2008 | Table 3. item 34 |
| Is there a standard definition for “normal” or “average” for the following? [Hearing only] | Knowledge | Familiarity with diagnostic criteria and tools | Sydlowski et al. 2022 | Supplementary materials Q 15 |
| How familiar are you with the following “normal” or “average” health metrics? | Knowledge | Familiarity with diagnostic criteria and tools | Sydlowski et al. 2022 | Table 2, Supp materials Q21 |
| For your patients 50 years of age or older who are diagnosed with hearing loss, which of the following sounds, if any, have they expressed missing due to hearing loss? | Knowledge | Knowledge of condition (aetiology, symptoms etc.) | Sydlowski et al. 2022 | Supplementary materials Q50 |
| Hearing loss is preventable | Knowledge | Knowledge of condition (aetiology, symptoms etc.) | Sydlowski et al. 2022 | Table 6, Supp materials Q39 |
| Does hearing loss negatively affect older persons’ quality of life? | Knowledge | Impacts and comorbidities | Danhauer et al. 2008, Johnson et al. 2008 | Table 2. item 17 |
| Hearing loss often causes difficulties for older patients’ relationships with their spouse and family | Knowledge | Impacts and comorbidities | Gilliver & Hickson, 2011 | Appendix A. item 7, Table 1 Q7 |
| Hearing loss in older patients can seriously impair independence | Knowledge | Impacts and comorbidities | Gilliver & Hickson, 2011 | Appendix A. item 13, Table 1 Q13 |
| Hearing loss impacts the quality of a patient's life | Knowledge | Impacts and comorbidities | Sydlowski et al. 2022 | Table 6, Supp materials Q41 |
| Hearing loss can lead to social isolation | Knowledge | Impacts and comorbidities | Sydlowski et al. 2022 | Table 6, Supp materials Q42 |
| Hearing loss can impact one's personal safety | Knowledge | Impacts and comorbidities | Sydlowski et al. 2022 | Table 6, Supp materials Q43 |
| Hearing is important to overall health | Knowledge | Impacts and comorbidities | Sydlowski et al. 2022 | Table 6, Supp materials Q38 |
| Hearing loss is Linked to increased risk for depression | Knowledge | Impacts and comorbidities | Sydlowski et al. 2022 | Supplementary table 1 |
| Hearing loss is Linked to an increased risk of falling | Knowledge | Impacts and comorbidities | Sydlowski et al. 2022 | Supplementary table 1 |
| Hearing loss is Linked to reduced income / job opportunity | Knowledge | Impacts and comorbidities | Sydlowski et al. 2022 | Supplementary table 1 |
| Hearing loss is Linked to increased risk for dementia | Knowledge | Impacts and comorbidities | Sydlowski et al. 2022 | Supplementary table 1 |
| Hearing loss is Linked to increased risk for type 2 diabetes | Knowledge | Impacts and comorbidities | Sydlowski et al. 2022 | Supplementary table 1 |
| Can about 40% of all hearing loss be treated medically? | Knowledge | Knowledge of treatments etc. | Danhauer et al. 2008, Johnson et al. 2008 | Table 2. item 20 |
| Many older patients only need to wear their hearing aids for specific activities/circumstances | Knowledge | Knowledge of treatments etc. | Gilliver & Hickson, 2011 | Appendix A. item 16, Table 2 Q16 |
| Hearing loss is treatable | Knowledge | Knowledge of treatments etc. | Sydlowski et al. 2022 | Table 6, Supp materials Q40 |
| “There’s no good answer for what to do if they have a hearing loss.” | Knowledge | Knowledge of treatments etc. | Zazove et al. 2017 | Table 2. mental model of the condition being prompted item 4 |
| Do about one in three people over 65 years of age have hearing loss? | Knowledge | Prevalence | Danhauer et al. 2008, Johnson et al. 2008 | Table 2. item 19 |
| Approximately what proportion of [your patients aged over 60] do you think would have a hearing loss (diagnosed or undiagnosed)? | Knowledge | Prevalence | Gilliver & Hickson, 2011 | Appendix A. item 2, in-text "Perceived Threat" p853 |
| Approximately what percentage of your older patients have sought help for hearing problems? | Knowledge | Prevalence | Gilliver & Hickson, 2011 | Appendix A. item 3 |
| Almost all of my older patients have hearing difficulties | Knowledge | Prevalence | Gilliver & Hickson, 2011 | Appendix A. item 11, Table 1 Q11 |
| Hearing loss is a normal part of aging | Knowledge | Prevalence | Sydlowski et al. 2022 | Table 6, Supp materials Q44 |
| Audiologists are well qualified to diagnose and manage nonmedical hearing and balance problems in the elderly. | Skills | Skills of other health professionals | Danhauer et al. 2008, Johnson et al. 2008 | Table 3. item 31 |
| I find it easy to discuss hearing rehabilitation and assessment with older patients | Beliefs about capabilities | Ease of discussion | Gilliver & Hickson, 2011 | Appendix A. item 18, Table 2 Q18 |
| "I don't have a script to address patients with hearing loss" | Beliefs about capabilities | Ease of discussion | Zazove et al. 2017 | Table 2. mental model of the condition being prompted item 1 |
| “I feel unprepared to answer patient’s questions about hearing loss and treatment options.” | Beliefs about capabilities | Familiarity with next steps | Zazove et al. 2017 | Table 2. mental model of the condition being prompted item 4 |
| It is easy/straightforward to refer older patients for hearing assessment/rehabilitation | Beliefs about capabilities | Familiarity with next steps | Gilliver & Hickson, 2011 | Appendix A. item 23, Table 2 Q23 |
| Hearing deteriorates with age, and does not always require amplification | Optimism | Unrealistic optimism | Gilliver & Hickson, 2011 | Appendix A. item 12, Table 1 Q12 |
| Screening for hearing loss in the elderly is a waste of resources, because there are few treatments for it. | Beliefs about consequences | Beliefs about hearing care outcomes/efficacy | Danhauer et al. 2008, Johnson et al. 2008 | Table 3. item 25 |
| Hearing aids are an effective rehabilitation tool for older patients | Beliefs about consequences | Beliefs about hearing care outcomes/efficacy | Gilliver & Hickson, 2011 | Appendix A. item 6, Table 2 Q6 |
| In general, my older patients achieve good outcomes with hearing aids | Beliefs about consequences | Beliefs about hearing care outcomes/efficacy | Gilliver & Hickson, 2011 | Appendix A. item 17, Table 2 Q17 |
| For many older patients, poor hearing is the result of increasing age and they would receive minimal benefit from amplification | Beliefs about consequences | Beliefs about hearing care outcomes/efficacy | Gilliver & Hickson, 2011 | Appendix A. item 24, Table 2 Q24 |
| For older people, the benefits of hearing aids often outweigh the potential disadvantages | Beliefs about consequences | Beliefs about hearing care outcomes/efficacy | Gilliver & Hickson, 2011 | Appendix A. item 26, Table 2 Q26 |
| Hearing aids have a lot to offer older patients with hearing loss | Beliefs about consequences | Beliefs about hearing care outcomes/efficacy | Gilliver & Hickson, 2011 | Appendix A. item 27, Table 2 Q27 |
| Do you think a HA can ameliorate a hearing problem? | Beliefs about consequences | Beliefs about hearing care outcomes/efficacy | Parving et al. 1996 | Table 2, appendix part 2 Q1 |
| Do you think a HA can ameliorate a hearing problem Watching TV? | Beliefs about consequences | Beliefs about hearing care outcomes/efficacy | Parving et al. 1996 | Table 2. item e), appendix part 2 Q2 |
| If you had a hearing problem would you consider a HA something beneficial? | Beliefs about consequences | Beliefs about hearing care outcomes/efficacy | Parving et al. 1996 | Table 4 |
| Can most nonmedical hearing loss be treated effectively with today’s hearing aids? | Beliefs about consequences | Beliefs about hearing care outcomes/efficacy | Danhauer et al. 2008, Johnson et al. 2008 | Table 2. item 21 |
| Approximately what proportion of your older patients do you think would reasonably benefit from hearing rehabilitation? | Beliefs about consequences | Beliefs about patient uptake of hearing care | Gilliver & Hickson, 2011 | Appendix A. item 3 |
| Many older patients find it difficult to adjust to using hearing aids | Beliefs about consequences | Beliefs about patient uptake of hearing care | Gilliver & Hickson, 2011 | Appendix A. item 8, Table 2 Q8 |
| Older patients will generally take/follow up the hearing rehabilitation advice given to them | Beliefs about consequences | Beliefs about patient uptake of hearing care | Gilliver & Hickson, 2011 | Appendix A. item 19, Table 2 Q19 |
| Do you think a HA can ameliorate a hearing problem for Face-to-Face conversation in quiet? | Beliefs about consequences | Social outcomes of hearing care | Parving et al. 1996 | Table 2. item a), appendix part 2 Q2 |
| Do you think a HA can ameliorate a hearing problem for Face-to-Face conversation in moderate background noise? | Beliefs about consequences | Social outcomes of hearing care | Parving et al. 1996 | Table 2. item b), appendix part 2 Q2 |
| Do you think a HA can ameliorate a hearing problem for minor group conversation? | Beliefs about consequences | Social outcomes of hearing care | Parving et al. 1996 | Table 2. item c), appendix part 2 Q2 |
| Do you think a HA can ameliorate a hearing problem at parties? | Beliefs about consequences | Social outcomes of hearing care | Parving et al. 1996 | Table 2. item d), appendix part 2 Q2 |
| Do you think a HA can ameliorate a hearing problem at the theatre, church etc.? | Beliefs about consequences | Social outcomes of hearing care | Parving et al. 1996 | Table 2. item f), appendix part 2 Q2 |
| If you did not use the HHIE and DHI previously, will you use them now to screen your elderly patients? | Intentions | Intention to change future behaviour | Danhauer et al. 2008, Johnson et al. 2008 | Table 1. item 11 |
| [Reason for not evaluating hearing loss] More pressing issues | Goals | Relative importance of other health conditions | Cohen et al. 2005 | Table 1 |
| Hearing loss is a low-priority health condition in the overall medical management of elderly patients | Goals | Relative importance of other health conditions | Danhauer et al. 2008, Johnson et al. 2008 | Table 3. item 27 |
| My older patients’ hearing is a high priority for me in relation to their overall health | Goals | Relative importance of other health conditions | Gilliver & Hickson, 2011 | Appendix A. item 5, Table 1 Q5 |
| [What factors sometimes make it difficult to discuss hearing with older patients?] 17 made reference to the lower priority of hearing in comparison to other health complaints | Goals | Relative importance of other health conditions | Gilliver & Hickson, 2011 | Appendix A. item 30, in-text "Referral Experiences" p854 |
| Thinking of your patients 50 years of age or older who need to manage multiple health conditions, please rank the following in order of importance to address by dragging them into the box on the right, where 1 is the most important health condition to manage | Goals | Relative importance of other health conditions | Sydlowski et al. 2022 | Table 3, Supp materials Q27 |
| Many commented that there are “more important” diseases to focus on; diabetes was an example mentioned multiple times. | Goals | Relative importance of other health conditions | Zazove et al. 2017 | In-text, p278 |
| [Indications for audiologist/otolaryngologist referral among respondents] Abnormal audiogram | Memory, attention and decision processes | Abnormal test results as a trigger for referral | Cohen et al. 2005 | Table 3 |
| [Indications for audiologist/otolaryngologist referral among respondents] Abnormal health questionnaire | Memory, attention, and decision processes | Abnormal test results as a trigger for referral | Cohen et al. 2005 | Table 3 |
| [Indications for audiologist/otolaryngologist referral among respondents] Abnormal tympanometry | Memory, attention, and decision processes | Abnormal test results as a trigger for referral | Cohen et al. 2005 | Table 3 |
| [Indications for audiologist/otolaryngologist referral among respondents] Abnormal tuning fork test | Memory, attention, and decision processes | Abnormal test results as a trigger for referral | Cohen et al. 2005 | Table 3 |
| [Reason for not evaluating hearing loss] Not enough time | Environmental context | Lack of time | Cohen et al. 2005 | Table 1 |
| [Reason for not evaluating hearing loss] No local otolaryngologist/audiologist | Environmental context | Lack of resources | Cohen et al. 2005 | Table 1 |
| I do not have time to do routine hearing and/or balance screenings for my elderly patients. | Environmental context | Lack of time | Danhauer et al. 2008, Johnson et al. 2008 | Table 3, item 30 |
| [Reason for not evaluating hearing loss] Evaluate only if patient reports problem | Social Influences | Patient or significant other initiates the discussion about hearing | Cohen et al. 2005 | Table 1 |
| [Indications for audiologist/otolaryngologist referral among respondents] Patient report of hearing loss | Social Influences | Patient or significant other initiates the discussion about hearing | Cohen et al. 2005 | Table 3 |
| What percent of your patients 50 years of age or older proactively ask you themselves or have a loved one ask you about their hearing health? (0-100%) | Social Influences | Patient or significant other initiates the discussion about hearing | Sydlowski et al. 2022 | Supplementary materials Q34 |
| It is very important to evaluate elderly patients’ hearing and/or balance if they complain about having problems. | Social influences | Patient or significant other initiates the discussion about hearing | Danhauer et al. 2008, Johnson et al. 2008 | Table 3. item 29 |
| What percent of your patients 50 years of age or older wait for you to initiate conversations about their hearing health? (0-100%) | Social Influences | Practitioner initiates the discussion about hearing | Sydlowski et al. 2022 | Supplementary materials Q35 |
| Older patients are concerned about the appearance of hearing aids (and the associated stigma of being seen wearing them) | Social Influences | Social stigma | Gilliver & Hickson, 2011 | Appendix A. item 10, Table 2 Q10 |
| Older patients would prefer to hide/deny any hearing impairment | Social Influences | Social stigma | Gilliver & Hickson, 2011 | Appendix A. item 15, Table 2 Q15 |
| It is a disadvantage for older patients that hearing aids make their hearing loss so obvious to other people | Social Influences | Social stigma | Gilliver & Hickson, 2011 | Appendix A. item 21, Table 2 Q21 |
| [What factors sometimes make it difficult to discuss hearing with older patients?] 35 participants’ responses related to feelings of stigma or reluctance to acknowledge hearing impairment | Social Influences | Social stigma | Gilliver & Hickson, 2011 | Appendix A. item 30, in-text "Referral Experiences" p854 |
